# Peripheral Inflammation and Cognitive Performance in Middle-Aged Adults With and Without Type 2 Diabetes: Results From the ENBIND Study

**DOI:** 10.3389/fnagi.2020.605878

**Published:** 2020-11-30

**Authors:** Adam H. Dyer, Louise McKenna, Isabella Batten, Karen Jones, Matthew Widdowson, Jean Dunne, Niall Conlon, Richard Reilly, Conor P. Woods, Desmond O’Neill, James Gibney, Nollaig M. Bourke, Sean P. Kennelly

**Affiliations:** ^1^Department of Age-Related Healthcare, Tallaght University Hospital, Dublin, Ireland; ^2^Department of Medical Gerontology, School of Medicine, Trinity College Dublin, Dublin, Ireland; ^3^Trinity Translational Medicine Institute, St. James’s Hospital, Dublin, Ireland; ^4^Department of Immunology, St. James’s Hospital, Dublin, Ireland; ^5^Robert Graves Institute of Endocrinology, Tallaght University Hospital, Dublin, Ireland; ^6^Trinity Centre for Bioengineering, Trinity College Dublin, Dublin, Ireland

**Keywords:** diabetes, cognition, dementia, inflammation, midlife

## Abstract

Midlife Type 2 Diabetes Mellitus (T2DM) is associated with a greater risk of dementia in later life. Peripheral inflammation and its impact on cognition is proposed as one of the pathological mechanisms mediating this link. However, studies have primarily focused on older individuals with established cognitive impairment and a long duration of T2DM. Importantly, knowledge of which individuals with midlife T2DM who are at greatest risk of later cognitive decline is lacking. We examined the cross-sectional relationship between serum levels of 8 pro-inflammatory markers (IL-1β, IL-6, TNF-α, IL-8, MCP-1, CXCL10, IL-12p70, CRP) and performance on a detailed neuropsychological assessment battery in middle-aged adults with uncomplicated T2DM (*N* = 89; 52 ± 8.1 years, 47% female) and matched healthy controls (*N* = 50; 52 ± 8.3 years, 59% female). Linear regression was used to analyze associations between serum markers and cognitive performance in the overall cohort, followed by a *T2DM^∗^protein concentration* interaction analysis to identify any T2DM-specific effects. We observed a significant T2DM-specific association between serum TNF-α levels and scores on the Paired Associates Learning (PAL) task (β: −3.16, SE: 1.32, *p* = 0.01, Std. Beta: −0.94), a task with significant working memory demands previously implicated in T2DM-related cognitive dysfunction. However, this did not persist on controlling for multiple testing. We provide exploratory evidence for a significant T2DM-specific relationship between serum TNF-α and memory performance. These findings require further replication and longitudinal analysis with the aim of selecting-out individuals with midlife T2DM at risk of future cognitive decline for potential preventative interventions.

## Introduction

Type 2 Diabetes Mellitus (T2DM) in midlife is associated with a greater risk of dementia in later life (Liebson et al., 1997a, b; Ott et al., 1999; Gudala et al., 2013; Jan Biessels and Despa, 2018). One of the most interesting aspects of this relationship is the age at which T2DM appears to be acting as a risk factor. Whilst elegant studies, such as the Atherosclerosis Risk in Communities (ARIC) study, have demonstrated the potent risk for dementia posed by midlife T2DM up to 20 years later (Rawlings et al., 2014; Gottesman et al., 2017; Knopman et al., 2018), in studies of those aged >65, T2DM does not appear to pose a similar risk for cognitive decline (Van den Berg et al., 2006). Thus, studying individuals with T2DM in midlife offers an important window into the cognitive risk posed by T2DM, as well as the putative pathological mechanisms explaining this link. Some of the hypothesized mechanisms mediating this link include peripheral inflammation, hyperglycemia, insulin resistance, perturbed functioning of the Hypothalamic-Pituitary-Adrenal (HPA) Axis in addition to a central role for vascular pathology and inflammation (Strachan, 2009; Reynolds et al., 2010; Strachan et al., 2011; Yaffe et al., 2011; Tuligenga et al., 2014; Ragy and Kamal, 2017).

There is significant evidence that both T2DM and dementia are diseases associated with significant systemic inflammation. In T2DM, localized inflammation in the pancreas, glucotoxicity, lipotoxicity, oxidative stress and endoplasmic reticulum stress results in activation of the innate immune system and pro-inflammatory macrophages (Donath and Sholeson, 2011). Such changes result in the secretion of pro-inflammatory cytokines such as Tumor Necrosis Factor α (TNF-α), Interleukin-1β (IL-1β), Interleukin-6 (IL-6) and Monocyte Chemoattractant Protein 1 (MCP-1) which are detectable at greater levels in the serum of those with T2DM in comparison to their non-diabetic counterparts (Donath and Sholeson, 2011; Donath, 2014). In assessing the link between cognitive impairment and T2DM, this peripheral inflammation may have a potentially important role in microglial priming, with peripheral cytokine gradients influencing pro-inflammatory reactions in the brain, causing or indeed accelerating cognitive dysfunction (Perry and Holmes, 2014). However, the relationship between peripheral inflammation and cognitive dysfunction has never been studied in those with midlife T2DM, at the exact age when T2DM is acting as a risk factor for later cognitive decline.

Many studies have noted elevated levels of pro-inflammatory cytokines in both T2DM (Spranger et al., 2003; King, 2018) and dementia (Guerreiro et al., 2007; Lee et al., 2018; Morgan et al., 2019). Similarly, studies in those without established cognitive impairment have noted an association between elevated pro-inflammatory cytokines in serum and future dementia risk (Engelhart et al., 2014). However, negative studies also exist, such as recent longitudinal analysis from the Mayo Clinic Study of Ageing (Wennberg et al., 2019), and the exact utility of serum pro-inflammatory cytokines as a marker of later cognitive decline across populations is yet to be fully established. Measurement of pro-inflammatory cytokines and its relationship to cognitive dysfunction may be particularly pertinent in those already at elevated risk of cognitive impairment, such as those with midlife T2DM, and may provide novel insight into shared pathogenesis.

In the Edinburgh Type 2 Diabetes Study, levels of three pro-inflammatory markers (IL-6, TNF-α and CRP) was associated with worse cognitive performance in 1,066 older adults with T2DM, many of whom had established T2DM-related complications (Marioni et al., 2010). Smaller studies have also demonstrated associations between serum levels of IL-1β (Tian et al., 2018) and cognitive function in older adults with T2DM, many of whom have T2DM-related complications. To date, no study has assessed the link between T2DM and cognitive performance in those with midlife T2DM before any micro- or macro-vascular complications have occurred, at the exact time when T2DM appears to be acting as a risk factor.

In the current study, we examined the relationship between a panel of serum pro-inflammatory markers in middle-aged adults with T2DM free from any diabetes-related complications or established cognitive impairment and cognitive performance examined using an extensive neuropsychological battery. We aimed to evaluate whether elevated levels of pro-inflammatory markers were associated with poorer cognitive performance in those with midlife T2DM, before any T2DM complications had been established.

## Methods

### Study Setting and Participant Recruitment

The current study was embedded within the ENBIND (Exploring Novel Biomarkers of Brain Health in Diabetes) Study. Ethical Approval was obtained from the Tallaght-St James’s Joint Research Ethics Committee [Reference: 2018/09/02/2018-10 List 34 (Liebson et al., 1997b)]. We recruited a cohort of middle-aged adults with T2DM free from any diabetes-related complications in addition to a cohort of healthy controls, matched for age, sex and educational attainment. Participants with T2DM were recruited from a specialist T2DM service in a tertiary referral hospital with matched controls recruited from the same catchment area by local advertisement.

### Inclusion and Exclusion Criteria

Inclusion criteria for the T2DM group included a confirmed diagnosis of T2DM and age at recruitment of 35–65 years of age. T2DM was defined as physician-diagnosed T2DM, attending a T2DM clinic in a tertiary referral center (Tallaght University Hospital). Exclusion criteria included: a diagnosis of non-type 2 Diabetes Mellitus (such as Type 1 Diabetes Mellitus or Gestational Diabetes Mellitus), evidence of microvascular (nephropathy, retinopathy or neuropathy) or macrovascular (history of cerebrovascular accident/transient ischemic attack, myocardial infarction, peripheral vascular disease) complications as per self-report or medical notes. Further, those with active depression, diagnosed psychiatric or neurological disorder, significant musculoskeletal, cardiorespiratory or other significant medical comorbidity (any disorder limiting an individual’s daily activities or with a known potential impact on cognitive function) were excluded. Controls of the same age and gender were free from any significant medical comorbidity as above. Controls were screened for previous history of T2DM, use of T2DM medication and by HbA1c outside the normal range.

As we wanted to study those free from existing cognitive impairment and active depression, participants with a score of <23 on the Montreal Cognitive Assessment (MoCA) or a score of >7 on the Center for Epidemiological Scale-8 (CESD-8) were excluded, based on normative data from the Irish population indicating potential cognitive impairment or elevated risk of current active depression (Kenny et al., 2013; Briggs et al., 2018).

### Clinical and T2DM Assessment

All participants underwent a clinical and T2DM assessment by a research physician. In addition to routine demographic characteristics, this included a comprehensive medical history, and in those with T2DM, an assessment for any T2DM-related complications. Educational attainment was categorized as primary, secondary (secondary school or high school) or tertiary (college/university or further education). A family history of dementia was categorized as a first degree relative with a diagnosis of dementia. Data was obtained on concurrent medication use by self-report. For those with T2DM, a T2DM history was taken including current treatment and duration of T2DM diagnosis. Additionally, T2DM participants were assessed for the presence of peripheral neuropathy using both physical examination and the Diabetic Neuropathy Symptom Score (DNSS score >1 indicating potential neuropathy) (Meijer et al., 2002). Those with a positive DNSS or physical examination from neuropathy were excluded from further participation. Hypertension/hypercholesterolemia was considered as having history of same, taking a regular anti-hypertensive/lipid-lowering agent or clinic blood pressure ≥140/90 mmHg (hypertension) or total/LDL cholesterol above the local laboratory reference range (hypercholesterolemia).

### Cognitive Assessment

Cognition was assessed in the first instance using the Montreal Cognitive Assessment (MoCA) as a general cognitive screener. Any participant with a MoCA score of <23 was excluded, consistent with normative Irish data (Kenny et al., 2013).

Detailed neuropsychological assessment was conducted using a customized computerized neuropsychological assessment battery (Lowe and Rabbit, 1998). The CANTAB battery lasted approximately 70 min in duration and assessed several neuropsychological domains, primarily memory, executive function and attention. The test was custom-designed for these areas which are known to be affected in T2DM-related cognitive impairment (Palta et al., 2014). All assessment and blood sampling took place during the hours of 9:00–17:00 in the research office, Department of Age-Related Healthcare, Tallaght University Hospital. The research office was well-lit, with minimal background noise. Before assessment, participants with T2DM were instructed to eat a normal meal and complete self-assessment of blood glucose before traveling to the study center to ensure glucose levels of >4.0 mmol/L prior to participation.

Briefly, the neuropsychological assessment battery consisted of the following tests:

(i) Paired Associates Learning (PAL): geometric patterns presented on screen, with participants having to memorize the locations of patterns with increasing difficulty. Performance assessed using the First Attempt Memory Score (PAL-FAMS; range: 0–20).
(ii) Spatial Working Memory (SWM): “tokens” are presented on screen and participants have to remember the locations in order to find additional “hidden tokens,” with increasing levels of difficulty. Performance is assessed using the Strategy (SWMS; range 2–12) score.
(iii) Pattern Recognition Memory Delayed (PRMD): participants are asked to memorize patterns and tested using a binary forced-choice paradigm after a 20-min delay. Percentage correctly remembered (PRM-PCD) is used to assess performance.
(iv) Reaction Time Task (RTT): assesses reaction time in milliseconds by asking a participant to press one of five circles one after another. Performance assessed by mean duration reaction time in milliseconds (RTT-MDRT), with increasing duration indicating poorer performance.
(v) One-Touch Stockings of Cambridge (OTS): assess executive function and attention, by moving colored balls inside stockings in the minimal number of moves in order to match a pattern seen on screen. The Percentage Solved on First Choice (PSFC) is used to assess performance.
(vi) Rapid Visual Processing (RVP): assesses ability to detect particular number sequences within a rapidly changing sequence of numbers. Performance is measured using a signal detection metric (termed A prime or A’) with poorer scores indicating poorer performance.

### Blood Sampling for Serum Pro-inflammatory Markers

Peripheral venipuncture was performed using standard aseptic technique by the research physicians at the study visit prior to cognitive assessment. Briefly, blood was collected in 6 mL Serum Clot Activator tubes and processed the same day as sampling. Samples were centrifuged and serum stored at −80°C until further analysis. Levels of C-Reactive Protein (CRP) were analyzed using a standardized high-sensitivity assay in the hospital laboratory. Levels of Interleukins-1β (IL-1β), 6 (IL-6), and 8 (IL-8) in addition to Tumor Necrosis Factor-α (TNF-α) were obtained using the multiplex ella ProteinSimple assay. Levels of Monocyte Chemoattractant Protein 1 (MCP-1), C-X-C Motif Chemokine Ligand 10 (CXCL10) and Interleukin-12p70 (IL-12p70) were analyzed using BD OptEIA Enzyme-Linked Immunosorbent Assay (ELISA) as per the manufacturer’s instructions.

### Statistical Analysis

All statistical analysis was carried out using STATA IC v15.0 (Texas, United States) and GraphPad Prism v8.0 (California, United States). Statistical significance was considered as *p* < 0.05. Descriptive statistics consisted of means/medians with standard deviations (SD)/interquartile ranges (IQR) as appropriate. Between-group statistics consisted of *t*-tests, wilcoxon rank sum tests and chi-square as appropriate to compare those with T2DM and healthy controls. Data was assessed for normality by visual inspection of histograms and Q-q plots. For peripheral immune markers, which were not normally distributed, a natural log (ln) transformation was used. Following this, data were further trimmed by removing observations greater than 3.5 standard deviations from the mean, consistent with extreme outliers.

In order to assess the association between serum markers of inflammation and cognitive function, we used linear regression with neuropsychological test performance as the dependent variable and the log-transformed immune marker/cytokine of interest as the independent variable. Models were adjusted for age, sex, Body Mass Index (BMI) and education level given the known importance of these variables to influence both cognition and serum markers of inflammation.

We firstly ran the models with the (natural) log transformed concentration as the independent variable in order to assess potential relationships between inflammation and cognitive function in the overall cohort independent of study group (T2DM and controls). In order to then test for T2DM-specific associations we then ran the models using a *T2DM^∗^ln(concentration)* interaction term as the predictor variable, controlling for age, sex and BMI. Results are reported in the first instance as Coefficients (β), Standard Errors (SE) and corresponding *p*-values. We additionally computed the standardized betas relating the regression models in order to aid interpretability of our findings.

Finally, we re-ran regression models adjusting for the above covariates (age, sex, BMI and education level) in addition to hypertension and hypersensitivity analysis to assess whether associations were affected by these vascular risk factors. No imputation was made for missing data. Correction for multiple testing was applied using the Bonferroni method.

## Results

### Study Participants

Of 140 participants screened, a single participant with T2DM was excluded for a MoCA score below the cut-off. No participants from either group were excluded based on CESD-8 score. Thus, 139 participants (52.3 ± 8.1 years of age, 50% female; 69/139) meeting the inclusion criteria were recruited to the current study, 89 of whom had a diagnosis of T2DM free from any diabetes-related complications in addition to 50 healthy controls. Notably, there was no significant difference in the groups in terms of age or sex, however, those with T2DM had a significantly higher mean BMI than the control group (Table 1). The groups did not differ in terms of other characteristics known to impact on cognitive function, such as educational attainment or family history of dementia (Table 1). In the T2DM group, the mean years since diagnosis was 5 (IQR: 2–11) and mean HbA1c in was 61.4 ± 19.8 mmol/mol indicating a relatively short duration of T2DM and good metabolic control. No controls were excluded based on use of T2DM medication or elevated HbA1c.

**TABLE 1:** Baseline characteristics of study participants by study group.

	Type 2 diabetes (*n* = 89)	Controls (*n* = 50)	Statistic
**Participant characteristic**			
Age	52 ± 8.1	53 ± 8.1	*t* = 0.08, *p* = 0.47
Sex, female	41 (47%)	29 (59%)	χ^2^ = 2.4, *p* = 0.12
Educational attainment *Primary Secondary Tertiary*	12 (14%) 60 (67%) 17 (19%)	3 (6%) 36 (72%) 11 (22%)	χ^2^ = 1.9, *p* = 0.39
Body mass index	32.3 ± 7.8	26.6 ± 3.3	*t* = −4.7, *p* < 0.001
Family history of dementia	17 (19%)	13 (26%)	χ^2^ = 1.2, *p* = 0.27
Hypertension	49 (55%)	5 (10%)	χ^2^ = 27.4, *p* < 0.001
Hypercholesterolemia	48 (54%)	6 (12%)	χ^2^ = 32.9, *p* < 0.001
HbA1c (mmol/mol)	61.4 ± 19.8	37 ± 3	*t* = −8.2, *p* < 0.001
Diagnosis duration	5 (2–11)	–	–
**Inflammatory markers**			
IL-1β (pg/mL)	0.65 (0–3.81)	1.21 (0.36–4.56)	*z* = −1.15, *p* = 0.25
IL-6 (pg/mL)	2.26 (1.61–5.29)	2.94 (1.86–4.07)	*z* = −0.76, *p* = 0.45
IL-8 (pg/mL)	20.3 (15.3–46.4)	33 (16.2–98.5)	*z* = −1.63, *p* = 0.11
TNF-α (pg/mL)	11.7 (8.33–15)	12.8 (9.8–16.2)	*z* = −0.58, *p* = 0.56
MCP-1 (pg/mL)	351 (257–528)	382 (245–616)	*z* = −0.67, *p* = 0.51
CXCL10 (pg/mL)	107 (62–189)	130 (68–193)	*z* = −0.86, *p* = 0.39
IL-12p70 (ng/mL)	4.14 (1.44–5.57)	2.63 (1.12–5.01)	*z* = −1.36, *p* = 0.17
CRP (ng/mL)	1.0 (0–2.0)	1.0 (1.0–4.0)	*z* = −1.67, *p* = 0.09
**Cognitive performance**			
Montreal cognitive assessment (MoCA)	29 (27–30)	29 (28–30)	*z* = 2.34, *p* = 0.02
Paired associates learning (First attempt memory score)	10 (7–14)	13 (9–15)	*z* = 1.79, *p* = 0.07
Spatial working memory (Strategy score)	9 (7–10)	9 (6–11)	*z* = 0.27, *p* = 0.79
Delayed pattern recognition (Percentage correct delayed)	77.8 (66.7–88.9)	83.3 (72.2–94.4)	*z* = 1.7, *p* = 0.09
Mean duration reaction time (ms)	409 (389–451)	403 (385–424)	*z* = −1.7, *p* = 0.09
One touch stockings of cambridge (Problems solved on first choice)	9 (7–11)	9 (7–12)	*z* = 0.83, *p* = 0.41
Rapid visual processing (A prime score)	0.89 (0.86–0.92)	0.90 (0.85–0.93)	*z* = 0.53, *p* = 0.60

*Results are presented as means with standard deviations (for age, Body Mass Index, HbA1c), medians with interquartile ranges (for diagnosis duration and inflammatory marker levels) and proportions for the remaining categorical data. IL-1β, Interluekin-1β; IL-6, Interluekin-6; IL-8, Interluekin-8; IL-6, IL-12p70 Interluekin-IL-12p70; TNF-α, Tumor Necrosis Factor α; MCP-1, Monocyte Chemoattractant Protein 1; CXCL10, CXC Containing Ligand 10; CRP, C-Reactive Protein.*

Of those with T2DM, one-fifth (19/89, 21.3%) were prescribed metformin-only, whilst a further fifth (17/89, 19.1%) were prescribed a Glucagon-Like Peptide 1 (GLP-1) analog either alone or in combination with metformin. Just under one third (26/89; 29%) were prescribed metformin in combination with a Sodium-Glucose Like Transporter 2 (SGLT2) Inhibitor or a Dipeptidy-Peptidase-4 (DPP4) Inhibitor in combination with metformin and a small minority (6/89; 6.7%) were prescribed insulin in addition to metformin.

### Pro-inflammatory Serum Markers and Cognition in Midlife T2DM

Overall, there was no significant difference in the levels of IL-1β, IL-6, IL-8, TNF-α, MCP-1, CXCL10, Il-12p70 or CRP between those with uncomplicated midlife T2DM and matched healthy controls. Full results are given in Table 1 with appropriate univariate statistics. Overall, 6 participants had missing data for IL-1β, 5 for IL-6, 5 for TNF-α, 3 for MCP-1, 1 for CRP. No participant was missing data for >2 markers. A single participant had missing data for the Pattern Recognition Memory Task, with no other missing cognitive data.

On analysis of the overall cohort (both T2DM and healthy controls), there was a significant association between increasing levels of CXCL10 and poorer performance on the Pattern Recognition Memory (Delayed) tasks (β:−3.31, SE: 1.53, *p* = 0.04, Std. Beta: −0.19). This association was not greater in those with T2DM (assessed via a T2DM^∗^natural log concentration interaction term). By analyzing results for potential T2DM-specific associations, we observed a significant association between increasing serum TNF-α and poorer performance on the Paired Associates Learning (PAL) task (β:−3.16, SE: 1.32, *p* = 0.01, Std. Beta: −0.94). These associations did not persist on controlling for multiple testing (Bonferroni correction).

On further analysis adjusting for hypertension and hypercholesterolemia, both the association between CXCL10 and Pattern Recognition Memory in the overall cohort (β:−3.81, SE: 1.61, *p* = 0.02, Std. Beta: −0.22) and the T2DM-specific association between TNF-a and poorer Paired Associates Learning persisted (β:−3.28, SE: 1.33, *p* = 0.02, Std. Beta: −0.98). There were no other T2DM-specific or overall associations seen on adjusting for hypertension and hypercholesterolemia. Again, these did not persist on controlling for multiple comparisons.

**FIGURE 1:**
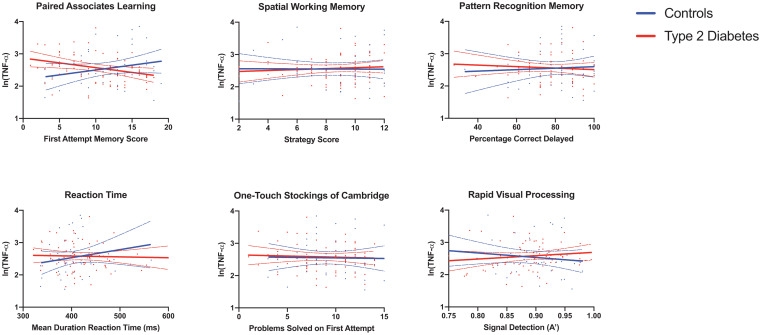
Associations between serum TNF-α and neuropsychological tests reveal a significant T2DM-specific association between elevated serum TNF-α and poorer performance on the paired associates learning task. Results for serum pro-inflammatory markers were log transformed. Linear regression, adjusted for age, sex and Body Mass Index, was used to assess the relationship between circulating pro-inflammatory cytokines and cognitive function. Individual data points, graphed by group are shown in addition to predicted lines of fit and 95% confidence intervals. There was a significant interaction between T2DM group status (midlife T2DM vs. controls) and the relationship between elevated serum TNF-α and poorer working memory performance. TNF-α, Tumor Necrosis Factor α; MCP-1, Monocyte Chemoattractant Protein 1; CXCL10, CXC Containing Ligand 10; CRP, C-Reactive Protein.

Full results are given for memory tasks in Table 2 and for reaction time, executive function and attention tasks in Table 3. No other T2DM-specific associations between peripheral inflammation and cognitive function were observed. The relationship between serum TNF-α and performance on all tasks of the neuropsychological assessment battery is presented graphically in Figure 1.

**TABLE 2:** Associations between peripheral inflammatory markers and neuropsychological tests of working and delayed memory in T2DM.

	Paired Associates Learning			Spatial Working Memory			Pattern Recognition Memory		
	β (SE)	P	Std. Beta	β (SE)	P	Std. Beta	β (SE)	P	Std. Beta
**ln (IL-1β)**									
Conc.	0.19 (0.24)	0.48	0.06	−0.21 (0.14)	0.88	−0.02	0.83 (0.85)	0.33	0.10
Conc.*T2DM	0.45 (0.48)	0.35	0.13	0.15 (0.28)	0.55	0.08	1.95 (1.71)	0.26	0.17
**ln (IL-6)**									
Conc.	0.06 (0.33)	0.86	0.02	0.04 (0.19)	0.82	0.02	−0.15 (1.15)	0.90	0.90
Conc.*T2DM	−0.69 (0.64)	0.28	−0.15	0.33 (0.38)	0.38	0.13	−0.73 (2.26)	0.75	−0.05
**ln (IL-8)**									
Conc.	−0.07 (0.24)	0.77	0.03	−0.01 (0.14)	0.95	−0.01	0.20 (0.83)	0.82	0.02
Conc.*T2DM	0.02 (0.47)	0.98	0.01	−0.03 (0.28)	0.90	−0.03	1.44 (1.67)	0.39	0.23
**ln (TNF-α)**									
Conc.	−0.26 (0.70)	0.72	−0.03	0.37 (0.41)	0.37	0.08	−0.02 (2.44)	0.99	−0.01
Conc.*T2DM	−3.16 (1.32)	0.01*	−0.94	−0.04 (0.80)	0.96	−0.02	−1.45 (4.77)	0.76	−0.13
**ln (MCP-1)**									
Conc.	0.42 (0.54)	0.44	0.07	−0.24 (0.31)	0.45	−0.06	−3.10 (1.92)	0.11	−0.15
Conc.*T2DM	0.42 (1.20)	0.73	0.28	−0.67 (0.70)	0.34	−0.76	0.64 (4.24)	0.88	0.13
**ln (CXCL10)**									
Conc.	−0.11 (0.43)	0.79	−0.02	−0.47 (0.24)	0.05	−0.16	−3.31 (1.53)	0.03*	−0.19
Conc.*T2DM	0.82 (0.93)	0.38	0.45	−1.02 (0.53)	0.06	−0.95	1.69 (3.38)	0.62	0.28
**ln (IL-12p70)**									
Conc.	−0.32 (0.35)	0.38	−0.08	0.24 (0.21)	0.26	0.10	−0.26 (1.28)	0.84	−0.02
Conc.*T2DM	−0.11 (0.79)	0.90	−0.09	0.33 (0.47)	0.49	0.48	0.96 (2.88)	0.74	0.25
**ln (CRP)**									
Conc.	−3.14 (2.19)	0.16	−0.17	−0.47 (0.33)	0.16	−0.16	−3.14 (2.19)	0.16	−0.17
Conc.*T2DM	0.25 (1.21)	0.84	0.05	−0.13 (0.71)	0.86	−0.04	8.32 (4.50)	0.07	0.44

*Results for serum pro-inflammatory markers were log transformed. Linear regression, adjusted for age, sex, Body Mass Index and educational attainment was used to assess the relationship between circulating pro-inflammatory cytokines and cognitive function. Results are presented as coefficients and standard errors in addition to standardized betas. IL-1β, Interluekin-1β; IL-6, Interluekin-6; IL-8, Interluekin-8; IL-6, IL-12p70 Interluekin-IL-12p70; TNF-α, Tumor Necrosis Factor α; MCP-1, Monocyte Chemoattractant Protein 1; CXCL10, CXC Containing Ligand 10; CRP, C-Reactive Protein.*

**TABLE 3:** Associations between peripheral inflammatory markers and neuropsychological tests of reaction time, executive function and attention in T2DM.

	Mean Reaction Time			Stockings of Cambridge			Rapid Visual Processing		
	β (SE)	P	Std. Beta	β (SE)	P	Std. Beta	β (SE)	P	Std. Beta
**ln (IL-1β)**									
Conc.	0.08 (0.06)	0.16	0.13	0.16 (0.27)	0.54	0.06	0.00 (0.00)	0.14	0.14
Conc.*T2DM	−0.03 (0.12)	0.77	−0.05	0.32 (0.61)	0.60	0.10	0.01 (0.01)	0.57	0.12
**ln (IL-6)**									
Conc.	0.08 (0.08)	0.31	0.09	−0.15 (0.25)	0.54	−0.06	−0.01 (0.00)	0.30	−0.14
Conc.*T2DM	0.04 (0.16)	0.79	0.04	−0.15 (0.49)	0.76	−0.04	0.01 (0.01)	0.42	0.12
**ln (IL-8)**									
Conc.	0.00 (0.01)	0.72	0.03	−0.32 (0.17)	0.07	−0.12	−0.00 (0.00)	0.40	−0.08
Conc.*T2DM	0.00 (0.11)	0.99	0.00	0.03 (0.35)	0.93	0.02	0.01 (0.01)	0.36	0.24
**ln (TNF-α)**									
Conc.	0.18 (0.02)	0.28	0.10	−0.11 (0.53)	0.83	−0.02	−0.02 (0.01)	0.12	−0.15
Conc.*T2DM	−0.08 (0.33)	0.82	−0.46	0.05 (1.03)	0.97	0.02	0.01 (0.02)	0.98	0.01
**ln (MCP-1)**									
Conc.	0.01 (0.13)	0.99	0.00	0.03 (0.40)	0.94	0.94	0.01 (0.01)	0.19	0.12
Conc.*T2DM	−0.30 (0.30)	0.32	−0.84	−1.21 (0.91)	0.19	−1.08	0.01 (0.02)	0.62	0.42
**ln (CXCL10)**									
Conc.	0.01 (0.10)	0.89	0.01	−0.56 (0.32)	0.08	−0.15	−0.00 (0.01)	0.92	−0.01
Conc.*T2DM	−0.18 (0.23)	0.43	−0.42	0.03 (0.69)	0.97	0.02	0.01 (0.01)	0.43	0.44
**ln (IL-12p70)**									
Conc.	−0.02 (0.09)	0.85	−0.02	−0.28 (0.27)	0.29	−0.10	−0.00 (0.01)	0.85	−0.02
Conc.*T2DM	−0.04 (0.20)	0.83	−0.29	−0.22 (0.61)	0.71	0.27	0.01 (0.01)	0.61	0.39
**ln (CRP)**									
Conc.	−0.14 (0.15)	0.36	−0.11	−0.62 (0.45)	0.17	−0.16	0.00 (0.01)	0.95	0.01
Conc.*T2DM	−0.13 (0.33)	0.69	−0.09	0.56 (0.95)	0.56	0.14	0.03 (0.02)	0.09	0.43

*Results for serum pro-inflammatory markers were log transformed. Linear regression, adjusted for age, sex, body mass index and educational attainment was used to assess the relationship between circulating pro-inflammatory cytokines and cognitive function. Results are presented as coefficients and standard errors in addition to standardized betas. IL-1β, Interluekin-1β; IL-6, Interluekin-6; IL-8, Interluekin-8; IL-6, IL-12p70 Interluekin-IL-12p70; TNF-α, Tumor Necrosis Factor α; MCP-1, Monocyte Chemoattractant Protein 1; CXCL10, CXC Containing Ligand 10; CRP, C-Reactive Protein.*

## Discussion

In the current study, we assessed the relationship between a panel of eight pro-inflammatory markers and cognitive function assessed using a detailed neuropsychological assessment battery in middle-aged adults with T2DM free from any diabetes-related complications and a matched cohort of healthy controls. We observed a significant association between circulating levels of CXCL10 and poorer memory performance in the overall cohort and a T2DM-specific association between increasing serum TNF-α levels and poorer cognitive performance on the Paired Associates Learning (PAL) task, although these did not persist on correction for multiple comparisons. Our findings add novel insight to the relationship between peripheral inflammation and cognition in midlife T2DM.

A notable finding from the current study is the lack of a significant difference in markers of peripheral inflammation between those with midlife T2DM and healthy controls. Whilst T2DM has been associated with an increase in circulating pro-inflammatory cytokines in some studies, more recent analyses have reported serum cytokine levels comparable to those without T2DM, particularly in those with good glycemic control and free from any diabetes-related complications (Gupta et al., 2017). The stringent inclusion criteria of the current study means that we selected for middle aged adults with a relatively short duration of diabetes, good glycemic control and free from any other T2DM complications. This was performed in order to assess for the earliest possible evidence linking peripheral inflammation and cognitive function in midlife T2DM, when T2DM appears to be acting as a risk factor for later cognitive decline and dementia in the first instance. Another reasons this for lack of between-group-difference in inflammatory markers includes the fact that many of the treatments prescribed for individuals in the current study, such as GLP-1 agonists (Hogan et al., 2013) and metformin (Cameron et al., 2016), have effects on circulating inflammatory markers.

The significant finding around increasing levels of serum TNF-α and poorer performance on the Paired Associates Learning (PAL) task is particularly interesting. This task is arguably one of the most demanding in the current study and has significant working memory demands, involving brain regions such as the prefrontal cortex, medial temporal lobe, hippocampus, basal ganglia and parietal cortex (Owen et al., 1995; Ryan et al., 2006). Interestingly, this task has been linked to elevated biomarkers of Alzheimer’s Disease, measured 10 years earlier in adults free from any cognitive impairment (Soldan et al., 2016). Many of the brain regions involved in performance on this task are known to be affected in individuals with T2DM (Moran et al., 2013). Further, previous studies have even demonstrated that improved metabolic control has been associated with better performance on this task in those with T2DM (Ryan et al., 2006). It is interesting that the association appeared to go in the opposite direction in healthy controls. The reasons for this are not clear, but are worthy of further replication and longitudinal analysis. Further follow up of this cohort will determine the exact relationship between TNF-α levels, performance on this task and later cognitive decline.

Whilst our findings are particularly novel in terms of the population studied (a middle-aged population free from T2DM complications), they are in-keeping with previous studies which have demonstrated cross-sectional associations of TNF-α levels with cognitive impairment in older adults with T2DM (Marioni et al., 2010). Both the current study and previous studies are limited by their cross-sectional nature, however, further longitudinal analysis of the current ENBIND study will assess the longitudinal relationships between cytokine levels and cognitive function in T2DM. Whilst there are some significant associations in longitudinal population-based studies between pro-inflammatory cytokines and cognitive decline (Engelhart et al., 2014), negative studies also exist. One of these, embedded within the Mayo Clinic Study of Aging, found no association between cytokine levels and global or domain-specific cognitive function (Wennberg et al., 2019). Thus, findings such as the current one warrant replication in further longitudinal cohorts of those with T2DM, as the potential association (like the one in the current study) may be specific to those with T2DM.

There are a variety of influences on the serum levels of pro-inflammatory cytokines not limited to age, sex, body mass index, concurrent medication use and medical comorbidity. Further, circadian rhythm may influence the level of cytokines in serum, with diurnal variations noted in major inflammatory cytokines. Whilst our study was conducted between working hours, we cannot out rule that variation in the time of sampling may have confounded our findings. It is also important to acknowledge that the validity of a once-off serum measurement of pro-inflammatory cytokines may be limited in the prediction of cognitive decline. More important may be trajectories of pro-inflammatory makers measured at multiple time-points. It may be that change in baseline levels of inflammation are more predictive of cognitive decline than once-off measurements. Similarly, it may be that more dynamic measurements of immune function are required (for instance *ex vivo* stimulation of immune cells in response to various pro-inflammatory stimuli or studies of cellular immunometabolism). Further longitudinal research, such as future longitudinal follow-up of the ENBIND cohort, should aim to address these questions.

One of the limitations of the main finding of the current study (around performance on the Paired Associates Learning task and serum measurements of TNF-α levels) is that there was a number of comparisons made in the current study. Our analysis was exploratory in nature and driven by the hypothesis that elevated levels of pro-inflammatory cytokines would be associated with poorer neuropsychological test performance. Whilst it may be argued that the significant finding around TNF-α and Paired Associates Learning performance is a by-product of multiple testing, it is notable that performance on this task has previously been noted in T2DM in addition to the fact that it assesses much of the same brain regions known to be impaired in T2DM on structural neuroimaging studies.

Another limitation of the current study may include the small number of participants. Whilst relatively small in comparison to the main previous study on cognitive function and inflammation in T2DM (Marioni et al., 2010), our study is unique in the population studied. Strict inclusion criteria, including age, lack of T2DM macro and microvascular complications meant that a large number of potential participants were not eligible to participate. The current study is notable in its inclusion of a very young population in comparison to previous studies in addition to the fact that T2DM participants were free from any significant micro or macrovascular complications of T2DM. Our study is unique in this regard and is the first such study in those with uncomplicated T2DM with a relatively short duration of T2DM. Previous studies, such as findings from the Edinburgh Type 2 Diabetes study, have been mainly carried out in adults older than those in the current study, with a longer duration of diabetes and the presence of T2DM related complications.

Finally, bias may have arisen in the current study due to different selection procedures between those with T2DM and healthy controls. Those with T2DM were recruited from a specialty clinic in a tertiary referral hospital, whilst control participants were recruited by local advertisement. These divergent selection procedures may have led to bias in the current study.

In light of the current findings, further longitudinal studies are warranted. Such studies would shed important light on the putative pathophysiological underlying the risk of cognitive impairment in midlife T2DM. Further, insight around which individuals are most at risk of cognitive decline may aid in selecting-out those with midlife T2DM who are at most risk of cognitive decline. This may be particularly important in the use of potential multi-domain preventative cognitive interventions in those with midlife T2DM, which whilst successful in certain populations, have been infrequently studied in those with T2DM (Dyer et al., 2020).

In conclusion, we assessed the cross-sectional relationship between eight serum pro-inflammatory markers and cognitive performance in middle-age adults with and without Type 2 Diabetes. We found a significant association between elevated serum TNF-α and poorer performance on the Paired Associates Learning task which was specific to individuals with T2DM in midlife, free from any diabetes-related complications. Our findings warrant further replication and longitudinal analysis of the association between pro-inflammatory cytokines and cognitive function in middle-aged adults with T2DM, at the window when T2DM is acting as a potent risk factor for the later development of dementia.

## Data Availability Statement

The datasets presented in this article are not readily available due to the potential for identification of study participants. A limited dataset may be requested from the corresponding author. Requests to access the datasets should be directed to AD, dyera@tcd.ie.

## Ethics Statement

The studies involving human participants were reviewed and approved by the Tallaght University Hospital/St James’s Hospital Joint Research Ethics Committee. The patients/participants provided their written informed consent to participate in this study.

## Author Contributions

AD, SK, and NB designed the protocol, conducted the current study and had oversight on all recruitment, assessment and analysis of laboratory parameters. LM recruited and assessed participants. IB, KJ, JD, and NC assisted with laboratory experiments. MW, CW, and JG assisted with participant recruitment. DO’N, RR, SK, NB, and AD assisted with concept and design of the study in addition to critically appraising the final manuscript. AD, NB, and SK manuscript writing and revision. All authors approved the final manuscript.

## Conflict of Interest

The authors declare that the research was conducted in the absence of any commercial or financial relationships that could be construed as a potential conflict of interest.
